# Lack of robust satellite cell activation and muscle regeneration during the progression of Pompe disease

**DOI:** 10.1186/s40478-015-0243-x

**Published:** 2015-10-28

**Authors:** Gerben J. Schaaf, Tom JM van Gestel, Esther Brusse, Robert M. Verdijk, Irenaeus FM de Coo, Pieter A. van Doorn, Ans T. van der Ploeg, WWM Pim Pijnappel

**Affiliations:** Molecular Stem Cell Biology, Department of Clinical Genetics, Erasmus MC University Medical Center, Rotterdam, The Netherlands; Department of Pediatrics, Erasmus MC University Medical Center, Rotterdam, The Netherlands; Center for Lysosomal and Metabolic Diseases, Erasmus MC University Medical Center, Rotterdam, The Netherlands; Department of Neurology, Erasmus MC University Medical Center, Rotterdam, The Netherlands; Department of Pathology, Erasmus MC University Medical Center, Rotterdam, The Netherlands

**Keywords:** Pompe disease, Satellite cells, Pax7, Muscle regeneration, Metabolic myopathy, Acid alpha glucosidase (GAA)

## Abstract

**Introduction:**

Muscle stem cells termed satellite cells are essential for muscle regeneration. A central question in many neuromuscular disorders is why satellite cells are unable to prevent progressive muscle wasting. We have analyzed muscle fiber pathology and the satellite cell response in Pompe disease, a metabolic myopathy caused by acid alpha-glucosidase deficiency and lysosomal glycogen accumulation. Pathology included muscle fiber vacuolization, loss of cross striation, and immune cell infiltration.

**Results:**

The total number of Pax7-positive satellite cells in muscle biopsies from infantile, childhood onset and adult patients (with different ages and disease severities) were indistinguishable from controls, indicating that the satellite cell pool is not exhausted in Pompe disease. Pax7/Ki67 double stainings showed low levels of satellite cell proliferation similar to controls, while MyoD and Myogenin stainings showed undetectable satellite cell differentiation. Muscle regenerative activity monitored with expression of embryonic Myosin Heavy Chain was weak in the rapidly progressing classic infantile form and undetectable in the more slowly progressive childhood and adult onset disease including in severely affected patients.

**Conclusions:**

These results imply that ongoing muscle wasting in Pompe disease may be explained by insufficient satellite cell activation and muscle regeneration. The preservation of the satellite cell pool may offer a venue for the development of novel treatment strategies directed towards the activation of endogenous satellite cells.

**Electronic supplementary material:**

The online version of this article (doi:10.1186/s40478-015-0243-x) contains supplementary material, which is available to authorized users.

## Introduction

Healthy skeletal muscle has a remarkable capacity to repair both minor and severe damage. Extensive muscle damage, such as caused by exercise or in muscular disease, requires a stem cell-mediated response. The muscle stem cells or muscle satellite cells are located at the myofiber periphery underneath the basal lamina [1]. Satellite cells are normally quiescent, but become rapidly activated after damage and proliferate to generate a large set of progeny that is capable of regenerating damaged myofibers by fusion. Recent elegant studies using mouse models that allow conditional depletion of the muscle stem cell pool showed that satellite cells are indispensible for muscle regeneration [2–5].

Genetic diseases affecting skeletal muscle pose a continuous challenge to the muscle regenerative system. More than 700 neuromuscular disorders are known [6]. While the molecular pathological processes behind the muscle diseases are diverse, in all cases the balance between muscle injury and regeneration is disturbed resulting in progression of muscle wasting. The central question is why the muscle regenerative program is incapable of efficiently repairing disease-induced muscle damage.

One hypothesis to explain this is depletion of the satellite cell pool due to continuous regeneration attempts. This idea has been derived from in vitro characterization of myoblasts from Duchenne Muscular Dystrophy (DMD) patients, which showed reduced proliferative capacity [7]. In agreement, studies using a mouse model for DMD (termed mdx) suggested the existence of a subpopulation of satellite cells that is exhausted in mdx mice [8]. However, electron microscopy and immunofluorescent analysis of skeletal muscle biopsies have shown that the number of satellite cells can be increased rather than exhausted in muscular dystrophies (including DMD, myotonic dystrophy, Limb Girdle Muscular Dystrophy type 2A (LGMD2A)) and inflammatory myopathies (including polymyositis, sporadic inclusion body myositis, and dermatomyositis) [9–13]. Besides increased proliferation, also increased satellite cell differentiation was observed in the above diseases as indicated by increased expression of myogenic lineage (MyoD, Myogenin) and muscle regenerative (embryonic Myosin Heavy Chain (eMyHC)) markers. The chronic satellite cell activation and inflammation may result in Transforming Growth Factor (TGF-β) signaling-mediated fibrosis at the cost of muscle repair [14].

A different group of neuromuscular disorders are the metabolic myopathies [6]. Recent findings suggest that metabolic changes can affect satellite cell function and skeletal muscle regeneration [15]. Here we have investigated the muscle regenerative response in Pompe disease (OMIM 232300). Pompe disease is caused by acid-alpha glucosidase (GAA) deficiency resulting in lysosomal glycogen accumulation in a variety of tissues, but its effect is most damaging in skeletal muscle (reviewed in [16]). The clinical spectrum of Pompe disease ranges from severely affected infants (the classic infantile form) to children and adults with a slower progressive form of Pompe disease (the non-classic or late onset form) [17–21]. The muscle pathology in Pompe disease is distinct from that in muscular dystrophies as fibrosis is not a prominent feature and immune responses are considered to be low. The pathological changes in skeletal muscle of Pompe patients are progressive and range from enlarged lysosomes in between myofibrils to completely vacuolized myofibers.

We have analyzed skeletal muscle biopsies from classic infantile patients and from patients with childhood and adult onset Pompe disease and compared the extent of muscle damage, satellite cell activation, immune cell infiltration, and muscle regenerative activity.

## Materials and methods

### Patients and control biopsies

Muscle biopsies were taken from the Vastus Lateralis using a standard open surgery or needle biopsy procedure as described previously [22] from Pompe patients, two DMD patients and control subjects. Control subjects were selected for which a progressive neuromuscular disorder was ruled out by medical history and muscle tissue sections showed normal histology on haematoxylin and eosin staining. The Ethical Committee of the Erasmus MC University Medical Center approved the use of the biopsies (MEC 2007–103). Written informed consent was obtained from all patients and control subjects or their legal guardians. All samples were frozen in N_2_-chilled isopentane and cryosectioned (5–10 μm). Medical Research Council (MRC) sumscores were calculated from the assessment of the strength of 26 muscle groups essentially as described [23].

### Histology

HE staining and Periodic Acid Schiff (PAS) staining were performed on tissue that was processed into glycolmetacrylate (GMA) [24]. Acid Phosphatase (AP) staining was performed on cryosections [25].

### Scoring of muscle damage

Scoring of muscle damage was based on a system previously described by [22]. All sections were evaluated by two researchers (RV, GS), who were blinded to the identity and clinical details of the patients. The level of disturbance of cross striation and vacuole density as discernable by light microscopy and PAS-staining intensity was determined for sections of each patient on a scale from 0–3. The scale for each category is provided in Table 1. The number of damaged fibers was expressed as percentage of total number of fibers present in the section. The overall muscle damage score was expressed as the sum score of cross striation, vacuole density and PAS intensity.

**Table 1:** Scoring of muscle damage based on pathology in muscle fibers

Score	Cross striation	PAS-positive staining	Vacuoles
0	Normal	None	None
1	>75 % normal	Little in most and/or all	Little in all and/or significant in some
2	25–75 % normal	Significant in all and strong in some; significant and/or strong in most	Significant in all and/or many in some
3	<25 % normal	Very strong in all	Many in all

### Immunohistochemistry

5–10 μm cryosections of patient and control biopsies were stored until analysis at −80 °C. Sections were thawed before staining. Primary antibodies were directed against CD68, clone KP1 (M0814; DAKO; 1:800), eMyHC (F1.652; DSHB; 1:150), Ki67 (Ab15580; Abcam; 1:50), Laminin (L9393; Sigma; 1:500 or LS-C96142; LS Bio; 1:500), MyoD (SC304; Santa Cruz; 1:200), Myogenin (M-225 ; Santa Cruz; 1:200), Pax7 (DSHB; 1:50), CD3 (2GV6; Ventana; ready to use), CD20Cy; clone L26; Dako, 1:400). Following the primary antibody incubation, sections were rinsed and incubated with biotin-conjugated anti-mouse antibody (BA-2000; Vector labs,1:50) and then with Alexafluor594-conjugated streptavidin (S11227; Life Technologies, 1:500). Sections incubated with rabbit primary antibodies were subsequently incubated with Alexafluor 488-conjugated goat-anti rabbit antibodies (A11307; Life Technologies,1:500). Finally chicken anti-Laminin was detected with Alexafluor647-conjugated goat anti-chicken antibodies (A21449; Life Technologies, 1:500). All sections were counterstained with Hoechst33258 (H3569; Life Technologies, 1:15000). The slides were mounted with Mowiol (475904; Calbiochem). For some biopsies, limited amounts of sections of sufficient quality were available for immunofluorescent analysis, and not all stainings could be performed for all patients.

### Image acquisition and analysis

For digital imaging complete histological sections were scanned on a Hamamatsu NanoZoomer 2.0 (Hamamatsu Photonics). Images were analyzed using NDP view software (NDP View 1.2.31 ENG, Hamamatsu Photonics). Sections for immunofluorescence were scanned on a Zeiss LSM700 (Carl Zeiss B. V., Sliedrecht, The Netherlands) using tile-scan modality. Image analysis was performed using Fiji (fiji.sc/Fiji).

### Statistical analysis

All data are expressed as means ± SD. Multiple groups were compared using a one way non-parametric ANOVA. Bonferroni correction was applied as Post-hoc test to adjust for multiple testing. Statistical significance was set at *P* <0.05. All calculations were performed using Graphpad 5.0 (Graphpad software, USA).

## Results

### Study design and skeletal muscle pathology

**Fig. 1:**
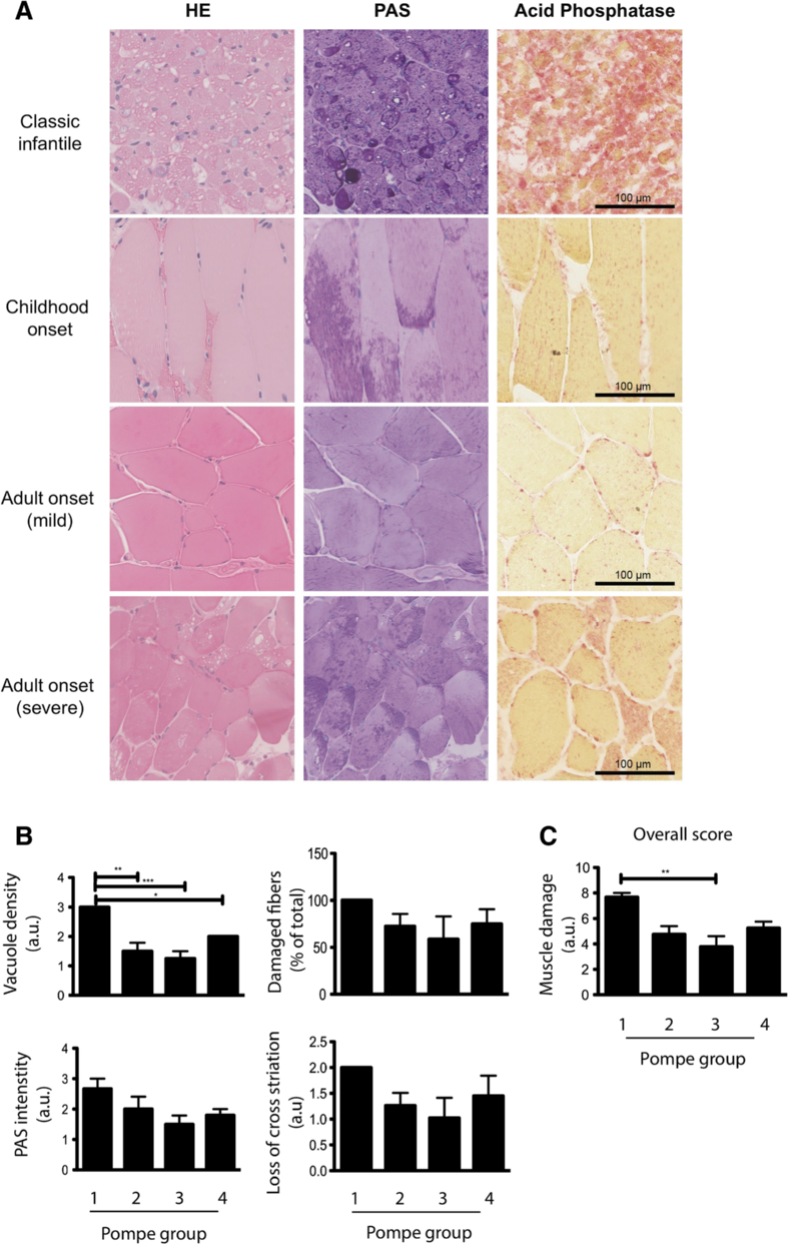
Skeletal muscle pathology of Pompe patients. Patients were divided in four groups as indicated based on disease onset and severity. **a** Representative examples of each group with HE (GMA), PAS (GMA), and acid phosphatase (frozen section) stainings of biopsies from the QF. **b** Biopsies were scored for vacuole density, % damaged fibers, PAS intensity, and % loss of cross striation for each patient group. Data are means +/− SD of three patients per group. **c** Scores of (**b**) were combined in an overall score for muscle damage. Group 1 *n* = 3, group 2 *n* = 2, group 3 *n* = 4, group 4 *n* = 5. Data are means +/− SD *: *p* < 0.05; **: *p* < 0.01. AU: arbitrary units

Biopsies of Pompe patients before the start of enzyme replacement therapy were taken from the Quadriceps Femoris (QF) and were used in this study. Patients were categorized into 4 groups based on age of disease onset and disease severity: (1) Classic infantile Pompe disease, with disease onset shortly after birth; (2) Childhood onset Pompe disease—disease onset ranging from 1 to 18 years; (3) Adult Pompe disease—mildly affected (>18 years old and <15 years disease symptoms); (4) Adult Pompe disease—severely affected (>18 years old and >15 years disease symptoms and requirement of walking aids and/or ventilator). Mildly affected adults (group 3) on average appeared younger as compared to severely affected adults (group 4) (Additional file 1: Table S1), although this was not significant. MRC sumscores were the lowest in the severely affected adult onset patient group, while these were higher in the childhood and mildly affected adult onset groups (Additional file 2: Figure S1). Histopathological findings are shown in Fig. 1a and quantified in Fig. 1b. HE staining was used to assess muscle damage, vacuolization, and cross striation. Damaged muscle, characterized by irregular shaped fibers and spaces in between the fibers were observed in all groups. This was also the case for loss of cross striation. Vacuolization, caused by extensive lysosomal pathology and muscle degeneration [26] was most extreme in biopsies from classic infantile patients and the least in mildly affected adults. PAS and acid phosphatase stainings were used to further evaluate enlarged lysosomes. Both stainings showed clear abnormalities for all patients examined. Classic infantile patients exhibited the most extensive PAS and acid phosphatase staining that was either localized or present throughout the entire muscle fiber. Similar but less severe staining was seen in the adult severely affected patients. Childhood onset and mildly affected adult patients lacked staining throughout the entire fibers but showed localized PAS- and acid phosphatase- positive areas. In GMA-fixed sections, no gross disruption of the sarcolemma was observed even in classic infantile patients. An overall scoring for muscle damage was performed based on the abnormalities described above (Fig. 1c). This shows an order of severity (from severe to less severe) of classic infantile (group 1), severely affected adults (group 4), childhood onset (group 2), and mildly affected adults (group 3).

### The satellite cell pool remains intact during disease progression

**Fig. 2:**
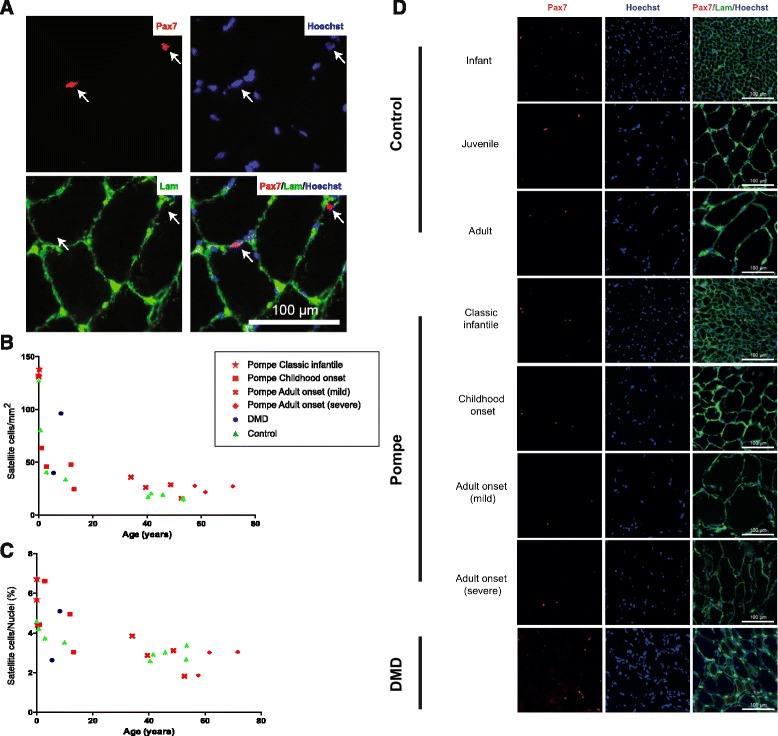
Satellite cell numbers remain unchanged and are independent of Pompe disease severity. **a** Identification of satellite cells (white arrows) by immunofluorescent analysis of Pax7 in human skeletal muscle biopsies. Upper left: Pax7; upper right: Hoechst; lower left: Laminin; lower right: merge. **b**, **c** Quantification of Pax7-positive cells in all Pompe biopsies examined in this study. Healthy control biopsies are included, as well as biopsies from two DMD patients. **b** Number of satellite cells (SCs)/mm^2^. **c** Percentage of SCs/nuclei. **d** Representative examples of each Pompe patient group, age-matched controls, and a DMD patient. Pompe patients: group 1 *n* = 3, group 2 *n* = 4, group 3 *n* = 4, group 4 *n* = 3. DMD patients: *n* = 2. Controls: infants *n* = 2, juveniles *n* = 2 and adult *n* = 5. For both Fig. 2b and c: Group 1 Pompe patients were statistically significant from other Pompe patient groups (*p* < 0.0001). Control infants were statistically significant from other controls (*p* < 0.0001). Other comparisons were not significantly different

Studies in rodents have shown that muscle repair is completely dependent on muscle satellite cells [2–4]. This raised the question how satellite cells respond to muscle pathology in Pompe disease. To determine whether the number of satellite cells is altered, satellite cells were identified and quantified in muscle biopsies using two criteria: positive nuclear staining for Pax7, a universal and sensitive marker of satellite cells [27, 28], and the characteristic location under the basal lamina (Fig. 2a). Quantification was performed by counting the number of satellite cells per surface area (Fig. 2b) and per total amount of nuclei (Fig. 2c). Representative stainings for patient groups and controls are presented in Fig. 2d. Both control and Pompe biopsies showed similarly high numbers of satellite cells at early ages of 1–2 years. Satellite cell numbers then decreased with age in both controls and Pompe patients without obvious differences. Two DMD patients of 5 and 8 years of age also showed normal satellite cell numbers. This indicated that despite severe disease progression, Pompe patients retain normal satellite numbers in the Quadriceps Femoris.

### Immune response

**Fig. 3:**
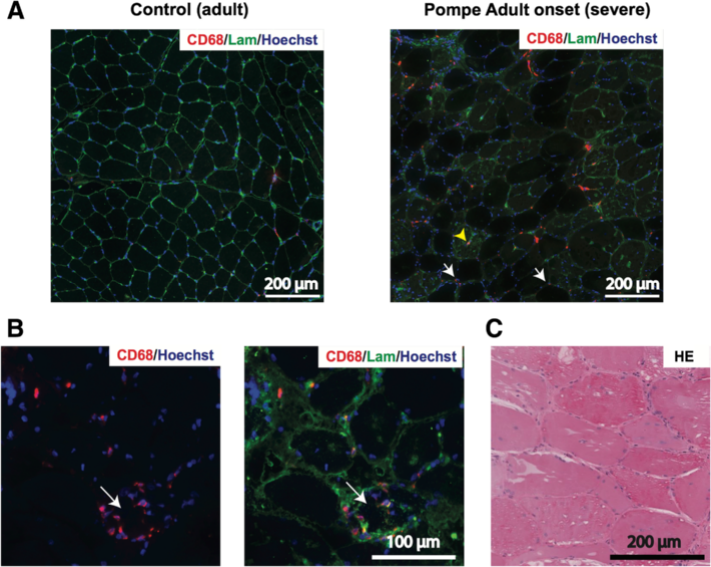
Macrophage infiltration in severely affected patients. **a** Immunofluorescent analysis of CD68 expression in biopsies from an adult control (left) or a severely affected adult onset Pompe patient (*right*). White arrows point to CD68-positive cells located between fibers, yellow arrowhead to CD68-positive cell invading a myofiber. **b** Detail of a necrotic fiber from the patient shown in Fig. 3a (*white arrow*) with CD68-positive cells around and inside the fiber. **c** GMA-fixed HE stained biopsy from a mildly affected Pompe patient

Myofiber necrosis is known to induce a rapid and transient activation of the innate immune system [29], which is essential for successful completion of muscle regeneration. Macrophages, both resident and recruited to damaged muscle, play diverse and critical roles in muscle regeneration in clearing necrotic debris and producing mitogens that drive satellite cell proliferation [30]. In neuromuscular disease, the ongoing muscle wasting process can affect the immune response with various outcomes. For example, a chronic immune response is thought to contribute to the development of muscle fibrosis in DMD [31]. To determine whether macrophages infiltrate skeletal muscle in Pompe disease, sections were stained for the pan-macrophage marker CD68. This showed the presence of CD68-positive cells in between muscle fibers (Fig. 3a, white arrows) and occasionally inside necrotic fibers (Fig. 3a, yellow arrowhead) from a severely affected adult onset patient which were absent in a control patient (Fig. 3a). Fig. 3b shows a detail of a biopsy from a severely affected patient with a necrotic fiber surrounded by and containing CD68-positive cells, suggesting active phagocytosis. In a GMA-fixed HE stained section of a biopsy from a mildly affected patient that showed substantial pathology, degenerating fibers with multiple macrophage-like nuclei inside the fiber, characteristic of early-stage necrosis, were identified, supporting the idea of phagocytosis (Fig. 3c). Biopsies with milder muscle fiber pathology did not show evidence of phagocytosis or elevated CD68 expression (data not shown). Adult patients with severe muscle pathology showed low numbers of end-stage necrotic fibers (<1 % of total fibers), whereas variable numbers of degenerating fibers with cell infiltration were observed ranging from 5 to 30 % of total fibers. The disrupted morphology of biopsies from classic infantile patients did not allow an analysis of CD68-positive cells around muscle fibers, however, numerous CD68-positive cells were observed (data not shown). No gross infiltration with CD20-positive B and CD3-positive T lymphocytes were detected in any patient group (<1–2 per section). These findings suggest that necrotic muscle fibers in Pompe patients are subject to macrophage-mediated phagocytosis in severely affected muscle biopsies. The repair of such damage is expected to require a satellite cell-mediated regenerative response.

### Compromised activation of satellite cells

**Fig. 4:**
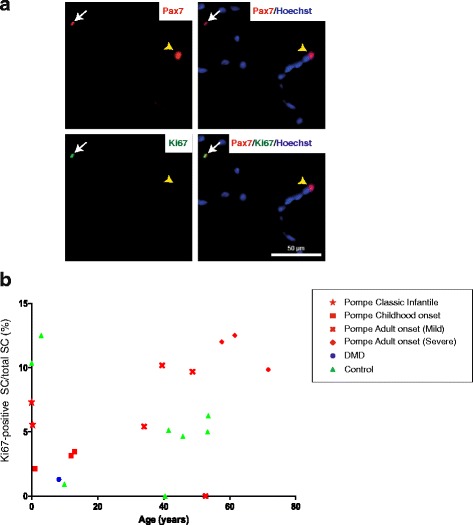
Unchanged satellite cell proliferation in Pompe disease. **a** Example of a proliferating (*arrow*) and a quiescent (*arrowhead*) satellite cell in a Pompe muscle biopsy. Cryosections were co-stained for Pax7 (*red*) to mark satellite cells and Ki67 (*green*) using immunofluorescence to assess active proliferation. Nuclei were stained with Hoechst (blue). White arrow indicates an activated satellite cell. The yellow arrowhead indicates a quiescent satellite cell. **b** Quantification of Ki67-positive satellite cells in Pompe patients (red symbols; different symbols per patient group), controls (green symbols), and a DMD patient (blue symbol). Pompe patients: group 1 *n* = 3, group 2 *n* = 3, group 3 *n* = 4, group 4 *n* = 3. DMD patient: *n* = 1. Controls: infants *n* =1, juveniles *n* = 2 and adults *n* = 5. There was no statistical difference between groups. The power to detect differences in Ki67-positive satellite cells was 0.8 (sample size 3, alpha 5 %, 2-sided equality, SD 5 %, delta 24). Delta of 24 is based on a previous report describing an increase of proliferating/activated satellite cells from ~0.5 % in healthy muscle to about 12 % after light resistance training [48]

The first step in activation of satellite cells, which are normally quiescent, involves cell proliferation. This result in temporary expansion of the satellite cell pool followed by differentiation to myoblasts. Triggers for activation include injury induced by exercise or disease. To determine to what extent satellite cells are activated in Pompe disease, biopsies were co-stained for Pax7 and Ki67, a marker of cell proliferation that is expressed during all phases of the active cell cycle but is absent in quiescent cells (Fig. 4a and Additional file 3: Figure S2). Ki67-positive satellite cells were detected in all four Pompe patient groups and varied from 2 to 12 % of the total Pax7-positive satellite cells (Fig. 4b), whereas the percentage of Ki67-positive nuclei was on average ~1 % (Additional file 4: Figure S3). Control biopsies showed a similar range of Ki67-positive satellite cells and Ki67-positive nuclei from infantiles to adults. The absolute numbers of Pax7/Ki67 double positive cells were low due to the low numbers of the satellite cells themselves hampering accurate quantification. Satellite cell activation by differentiation into the myogenic lineage was examined using stainings of MyoD and Myogenin. While MyoD and Myogenin-positive satellite cells could be detected in human (Additional file 5: Figure S4) and mouse (data not shown) muscle biopsies, their frequency was too low in all patient groups and controls (0–1 per biopsy) to allow quantitative analysis. Taken together, satellite cells in Pompe disease show low levels of activation that are indistinguishable from controls, and no disease-induced satellite cell response was observed.

### Muscle regeneration

**Fig. 5:**
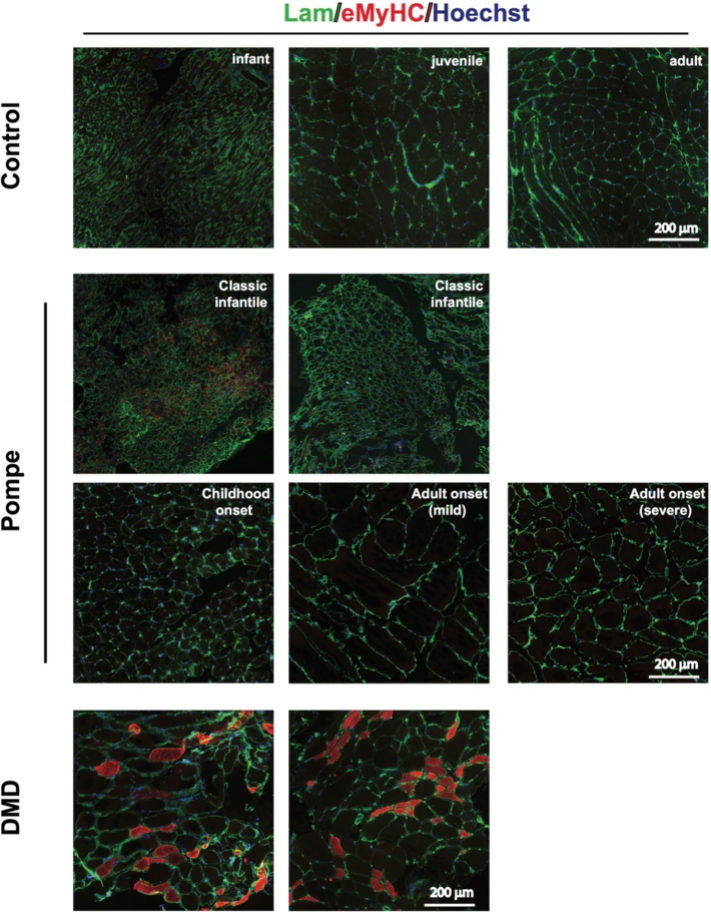
Impaired muscle regeneration in Pompe patients. Immunofluorescent analysis of embryonic myosin heavy chain (eMyHC in red) expression in to detect actively regenerating myofibers. Muscle sections were co-stained for Laminin (*in green*) to visualize the fiber outline, nuclei were stained with Hoechst (*in blue*). Representative examples are shown. Two examples from classic infantile Pompe patients and DMD patients are derived from different patients

The lack of satellite cell activation predicted that muscle regenerative activity is low in Pompe disease. To test this, eMyHC stainings were performed. eMyHC is expressed during normal muscle development in the embryo as well as transiently during active muscle repair postnatally [32]. eMyHC expression in postnatal skeletal muscle is a strong indicator of an active regeneration response. Biopsies from classic infantile patients showed weak (Fig. 5, left sections) to no detectable eMyHC (Fig. 5, right section) staining. eMyHC staining was undetectable in all childhood onset, mildly affected adult onset, and severely affected adult onset Pompe patients examined as well as in control biopsies (Fig. 5). In contrast, eMyHC was strongly expressed in DMD patients (Fig. 5). This underscored the absence of a robust muscle regenerative response in Pompe disease. However, GMA-fixed HE stained biopsies from the most severely affected adult patients showed some fibers with an array of centrally located nuclei inside muscle fibers (Additional file 6: Figure S5), suggesting that a weak regenerative response is present in strongly affected muscles.

## Discussion

### Satellite cell numbers are neither increased nor exhausted in Pompe disease

We have shown that the numbers of satellite cells in skeletal muscle biopsies from Pompe patients are similar to controls. The number of satellite cells is known to be age-dependent with a ratio of 12 % (satellite cells/myonuclei) at 1–2 years of age, followed by a decline to about 2 % in adults [33]. Pompe patients and controls followed a similar age-dependent pattern. This finding is surprising given the extensive muscle pathology, especially in classic infantile patients. These patients showed strongly disrupted myofiber organization with loss of cross striation, widespread glycogen accumulation, and numerous vacuoles, as described previously [22, 26, 34, 35]. Distinct pathology was seen in muscle biopsies from clinically severely affected adult patients and in some of the childhood onset patients. A priori, a satellite cell response to Pompe disease was anticipated. Based on the level of muscle damage, a possible scenario was exhaustion of the satellite cell population as a result of continuous cycles of regeneration, as has been proposed for DMD patients. Another scenario would be an increase in satellite cell numbers due to satellite cell activation resulting in increased proliferation, as seen in a number of neuromuscular disorders including dystrophies and inflammatory myopathies [9–13]. These possible scenarios are not necessary mutually exclusive and may be dependent on the disease severity and age at which the satellite status is examined. Here, we analyzed the satellite cell response for Pompe patients with various degrees of disease severity and different ages in order to cover these possible scenarios. This showed that during all disease stages including in classic infantile patients and in severely affected adult patients at advanced age (around 70 years), the satellite cell population remained intact, arguing against satellite cell exhaustion in Pompe disease.

### Impaired activation of satellite cells in Pompe patients

Both satellite cell proliferation, monitored from the percentage of Ki67-positive satellite cells, and differentiation, indicated by the number of MyoD- and Myogenin-positive cells, were similar to controls for all Pompe patient groups. This indicated a failure of a robust satellite cell response in Pompe disease irrespective of disease severity. A previous report described low levels of satellite cell activation in classic infantile Pompe patients monitored 1 year after enzyme replacement therapy [34]. Two patients that showed a good histological response to enzyme replacement therapy showed the lowest activation levels. It would be interesting to compare the effect of enzyme replacement therapy by comparing baseline and treated samples in future studies.

An important question is whether the failure of satellite cell activation in Pompe disease is caused by the absence of an activating signal or the presence of an inhibitory factor. In muscle injury induced experimentally or by exercise, sarcolemmal damage triggers satellite cell activation by disturbing the niche of the satellite cell in between the sarcolemma and basal lamina. In Pompe disease, no gross disruption of the sarcolemma can be observed even in classic infantile patients, as shown using electron microscopy [26] and light microscopy (provided glutaraldehyde-based fixation of biopsies). This would argue for reduced presence of a satellite cell-activating signal in Pompe disease. However we cannot rule out more subtle damage to the sarcolemma that has not been detected using these methods.

Subsequent signals involved in both activation and inhibition of muscle regeneration are derived from the immune system. Following injury, a transient immune response triggers satellite cell activation and muscle regeneration. However, chronic immune responses can potently inhibit muscle regeneration and induce TGFβ-signaling-mediated muscle fibrosis as is the case for the muscular dystrophies. An immune response in Pompe patients is a hereto understudied factor considering the paucity of published reports on this aspect. One previous report has also detected inflammatory cells in skeletal muscle of an adult Pompe patient [36], but the cells have not been further characterized. At the molecular level, microarray analysis of genome wide mRNA expression patterns indicate elevated expression levels of several genes involved in immune regulation in skeletal muscle biopsies from classic infantile patients [37]. In the current study, infiltration of skeletal muscle with CD68-positive cells was observed in severely affected Pompe patients and suggested the presence of macrophages involved in phagocytosis of degenerating muscle fibers. It remains to be determined how these macrophages affect satellite cell activation or inhibition, and to what extent glycogen accumulation in macrophages from Pompe patients [38, 39] plays a role in this process.

Another factor that may contribute to the deregulation of satellite cell activation is impaired autophagy. It has been suggested that hematopoietic stem cells depend on autophagy as energy source for proper activation [40], and recent results also indicate a similar role for autophagy in satellite cell activation [15]. Interestingly, autophagy in skeletal muscle is impaired in Pompe disease [41, 42], warranting further investigation on its role in modulating satellite cell activation.

### A venue for the development of novel treatment strategies

The positive news for Pompe patients is that their satellite cell pool is intact and not exhausted. This may open novel treatment options targeted at enhancement of satellite cell activation and improved muscle regeneration. A prerequisite for successful implementation of such an approach is that the intrinsic properties of satellite cells in Pompe patients are intact. Electron microscopy studies of satellite cells from Pompe patients showed normal morphology [26]. Studies focused on the functional characteristics of satellite cells in muscle of Pompe patients are required to fully address this subject. Several factors have been shown to be capable of enhancing satellite cell activation, including growth factors, small molecules that modulate signaling pathways, immune modulation, and physical exercise. In recent studies we and others have shown that controlled exercise is well tolerated by Pompe patients [43–47].

## Conclusions

In conclusion, despite the extensive muscle damage satellite cells do not become robustly activated during Pompe disease progression. In line with this, the regenerative response in skeletal muscle of classic infantile Pompe patients was weak and absent in childhood- and adult-onset Pompe patients. The preservation of the satellite cell pool may offer a venue for the development of novel treatment strategies directed towards the activation of endogenous satellite cells.

## Additional files

Additional file 1: Table S1. Age range of patients used in this study. (PDF 175 kb) Additional file 2: Figure S1. MRC sumscores of non-classic patients per group. Classic infantile patients were not assessed due to their age. The figure depicts mean values (horizontal lines) and standard deviations, respectively. Pompe patients: group 2 *n* = 3, group 3 *n* = 6, group 4 *n* = 6. ***p* < 0.001. (PDF 79 kb) Additional file 3: Figure S2. Examples of immunofluorescent Ki67 stainings. Left: Human tonsil was stained as positive control and shows multiple Ki67-positive nuclei. Right: Example of Pax7/Ki67 double staining of skeletal muscle from a Pompe patient to identify quiescent (arrowhead) and activated (arrow) satellite cells. (PDF 325 kb) Additional file 4: Figure S3. Unchanged satellite cell proliferation in Pompe disease. Cryosections from Pompe muscle biopsies were co-stained for Pax7 to mark satellite cells and Ki67 (see Fig.4) using immunofluorescence to assess active proliferation. Nuclei were stained with Hoechst (blue; Fig.4). The figure depicts the quantification of Ki67-positive satellite cells in Pompe patients (red symbols; different symbols per patient group), controls (green symbols), and a DMD patient (blue symbol). Data are expressed as % Ki67-positive satellite cells per total nuclei. Pompe patients: group 1 *n* = 3, group 2 *n* = 3, group 3 *n* = 4, group 4 *n* = 3. DMD patient: *n* = 1. Controls: infantile *n* =1, juvenile *n* = 2, adult *n* = 5. There were no statistical differences between patient and control groups. (PDF 963 kb) Additional file 5: Figure S4. Example of MyoD (red, arrow) and Myogenin (green, arrow) immunofluorescent stainings. These markers could hardly be detected in Pompe muscle and the figure shows an example of a skeletal muscle biopsy from a DMD patient as positive control. Nuclei were stained with Hoechst (blue). (PDF 428 kb) Additional file 6: Figure S5. Examples of muscle fibers that show evidence of recent muscle regeneration. The arrows point to centrally located arrays of nuclei. The two photographs were derived from a muscle biopsy of a severely affected Pompe patient. (JPEG 4375 kb)
