# Supplementary material for: Lack of robust satellite cell activation and muscle regeneration during the progression of Pompe disease
Source: Acta Neuropathol Commun. 2015 Oct 28;3:65. doi: 10.1186/s40478-015-0243-x (PMC4625612; doi:10.1186/s40478-015-0243-x)
Supplement: Additional file 1: Table S1. — Age range of patients used in this study. (PDF 175 kb) [file 40478_2015_243_MOESM1_ESM.pdf]

Supplemental Table 1: Description of patient and control samples

Pompe patient group	n	age range (years)	Mean age
**1**	3	0.01-0.3	0.13
**2**	4	1.06-13.09	7.2
**3**	6	34.08-62.87	48.6
**4**	6	33.46-71.68	56.6
**DMD patients**	**n**	**age range (years)**	**Mean age**
	2	5.5-8.25	6.9
**control group**	**n**	**age range (years)**	**Mean age**
**infants**	3	0.01-0.67	0.2
**juveniles**	3	2.92-16.16	9.7
**adults**	5	40.44-53.5	46.9
