# Supplementary figures and images for: Lack of robust satellite cell activation and muscle regeneration during the progression of Pompe disease
Source: Acta Neuropathol Commun. 2015 Oct 28;3:65. doi: 10.1186/s40478-015-0243-x (PMC4625612; doi:10.1186/s40478-015-0243-x)

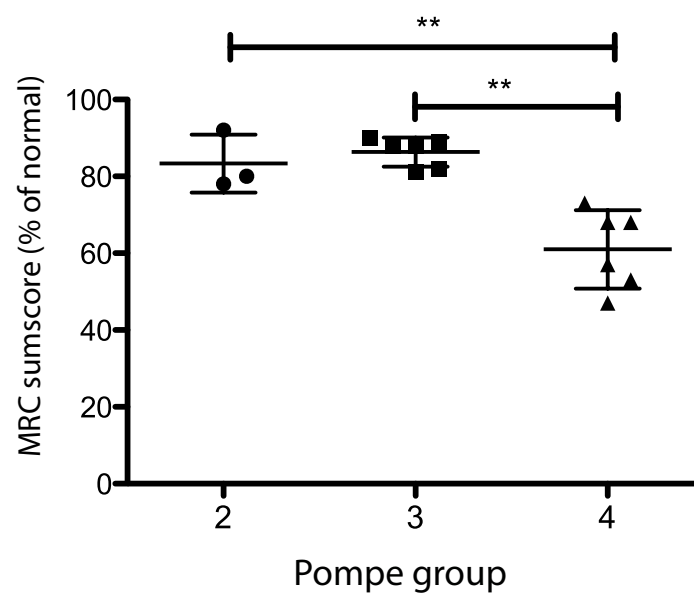

Supplement: Additional file 2: Figure S1. — MRC sumscores of non-classic patients per group. Classic infantile patients were not assessed due to their age. The figure depicts mean values (horizontal lines) and standard deviations, respectively. Pompe patients: group 2 n = 3, group 3 n = 6, group 4 n = 6. **p < 0.001. (PDF 79 kb) [file 40478_2015_243_MOESM2_ESM.pdf]

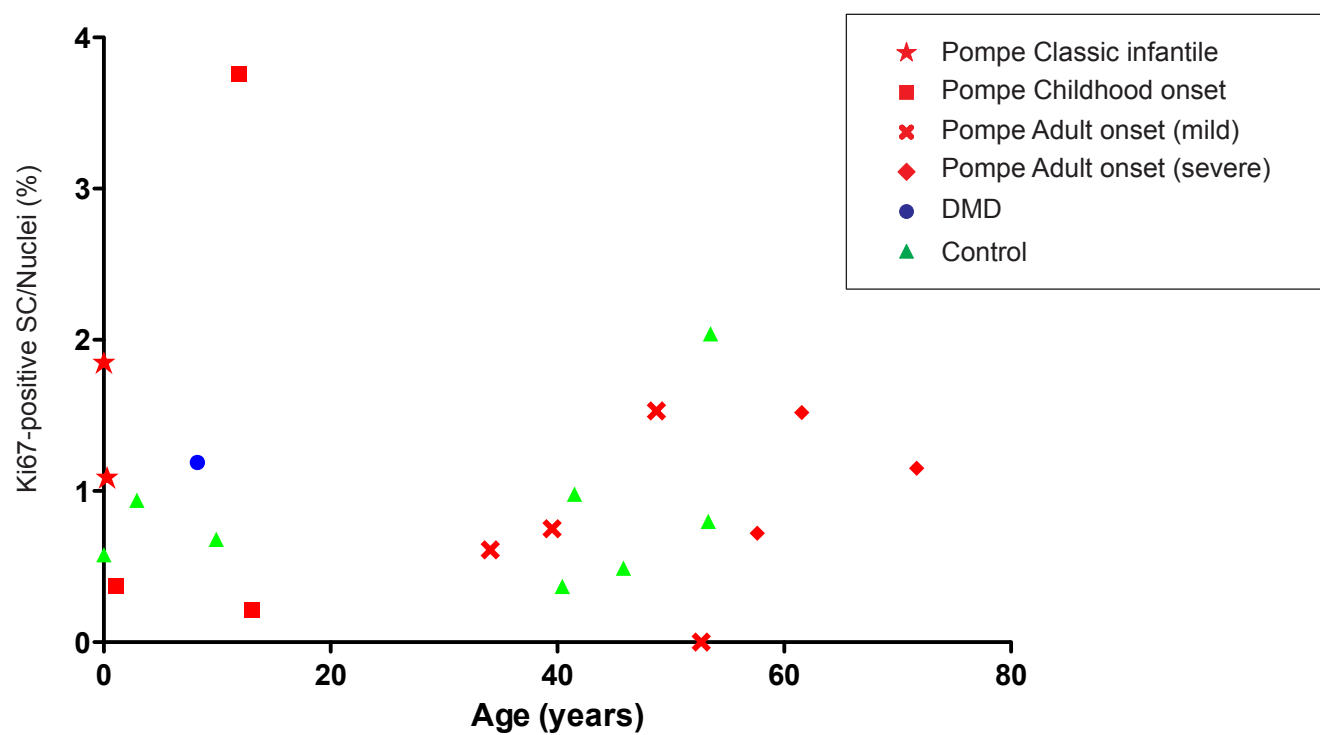

Supplement: Additional file 3: Figure S2. — Examples of immunofluorescent Ki67 stainings. Left: Human tonsil was stained as positive control and shows multiple Ki67-positive nuclei. Right: Example of Pax7/Ki67 double staining of skeletal muscle from a Pompe patient to identify quiescent (arrowhead) and activated (arrow) satellite cells. (PDF 325 kb) [file 40478_2015_243_MOESM3_ESM.pdf]

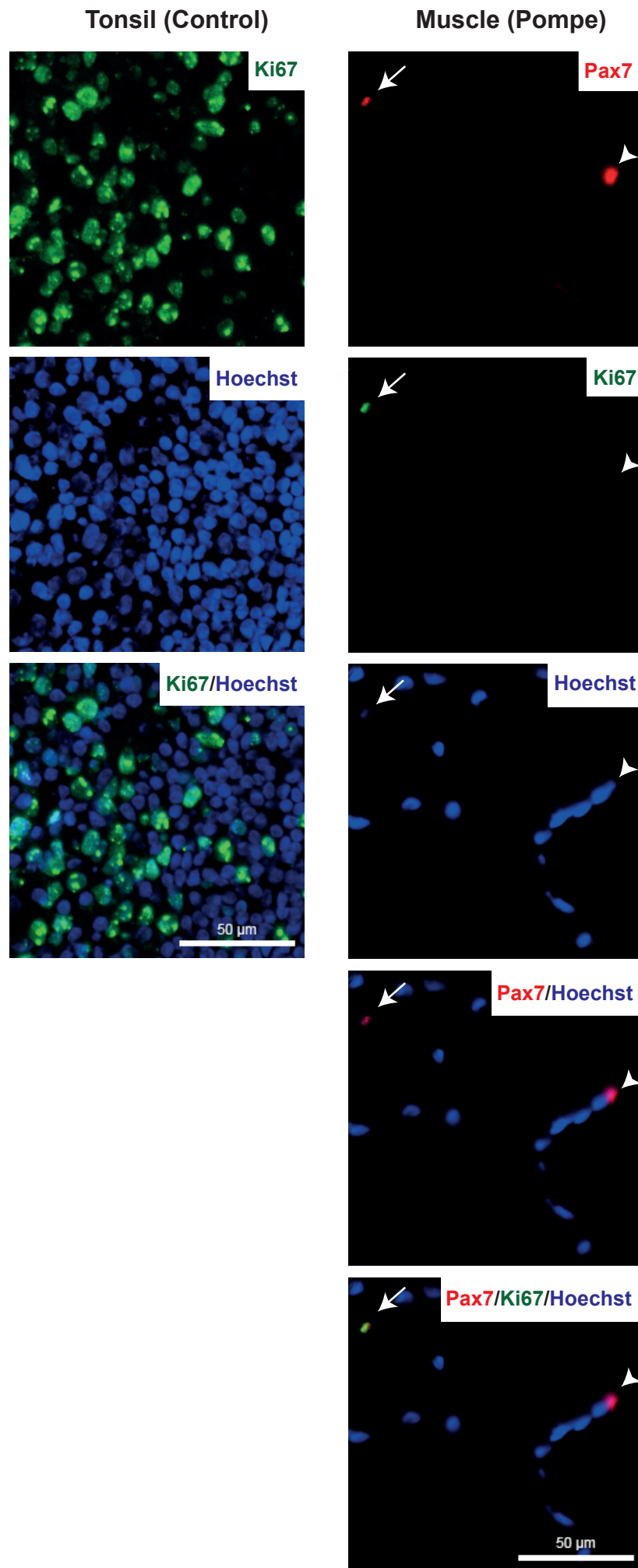

Supplement: Additional file 4: Figure S3. — Unchanged satellite cell proliferation in Pompe disease. Cryosections from Pompe muscle biopsies were co-stained for Pax7 to mark satellite cells and Ki67 (see Fig.4) using immunofluorescence to assess active proliferation. Nuclei were stained with Hoechst (blue; Fig.4). The figure depicts the quantification of Ki67-positive satellite cells in Pompe patients (red symbols; different symbols per patient group), controls (green symbols), and a DMD patient (blue symbol). Data are expressed as % Ki67-positive satellite cells per total nuclei. Pompe patients: group 1 n = 3, group 2 n = 3, group 3 n = 4, group 4 n = 3. DMD patient: n = 1. Controls: infantile n =1, juvenile n = 2, adult n = 5. There were no statistical differences between patient and control groups. (PDF 963 kb) [file 40478_2015_243_MOESM4_ESM.pdf]

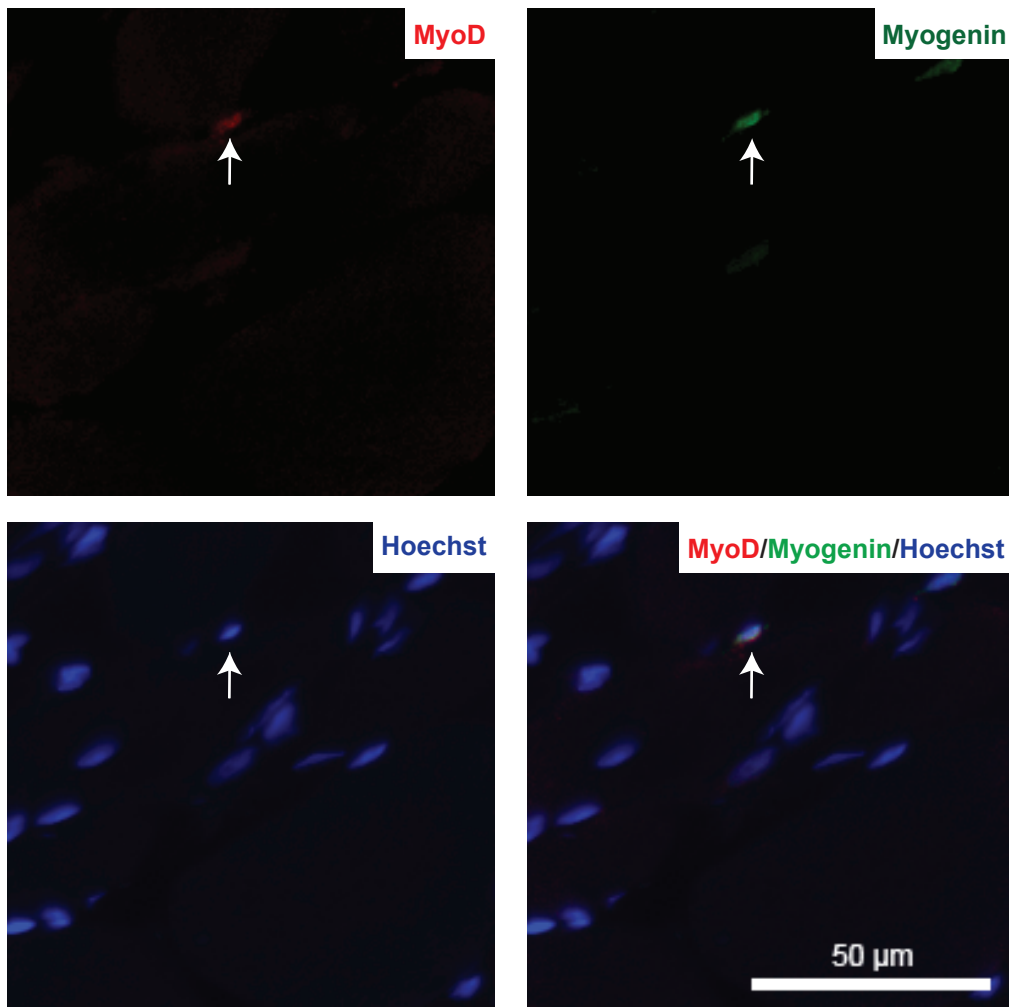

Supplement: Additional file 5: Figure S4. — Example of MyoD (red, arrow) and Myogenin (green, arrow) immunofluorescent stainings. These markers could hardly be detected in Pompe muscle and the figure shows an example of a skeletal muscle biopsy from a DMD patient as positive control. Nuclei were stained with Hoechst (blue). (PDF 428 kb) [file 40478_2015_243_MOESM5_ESM.pdf]

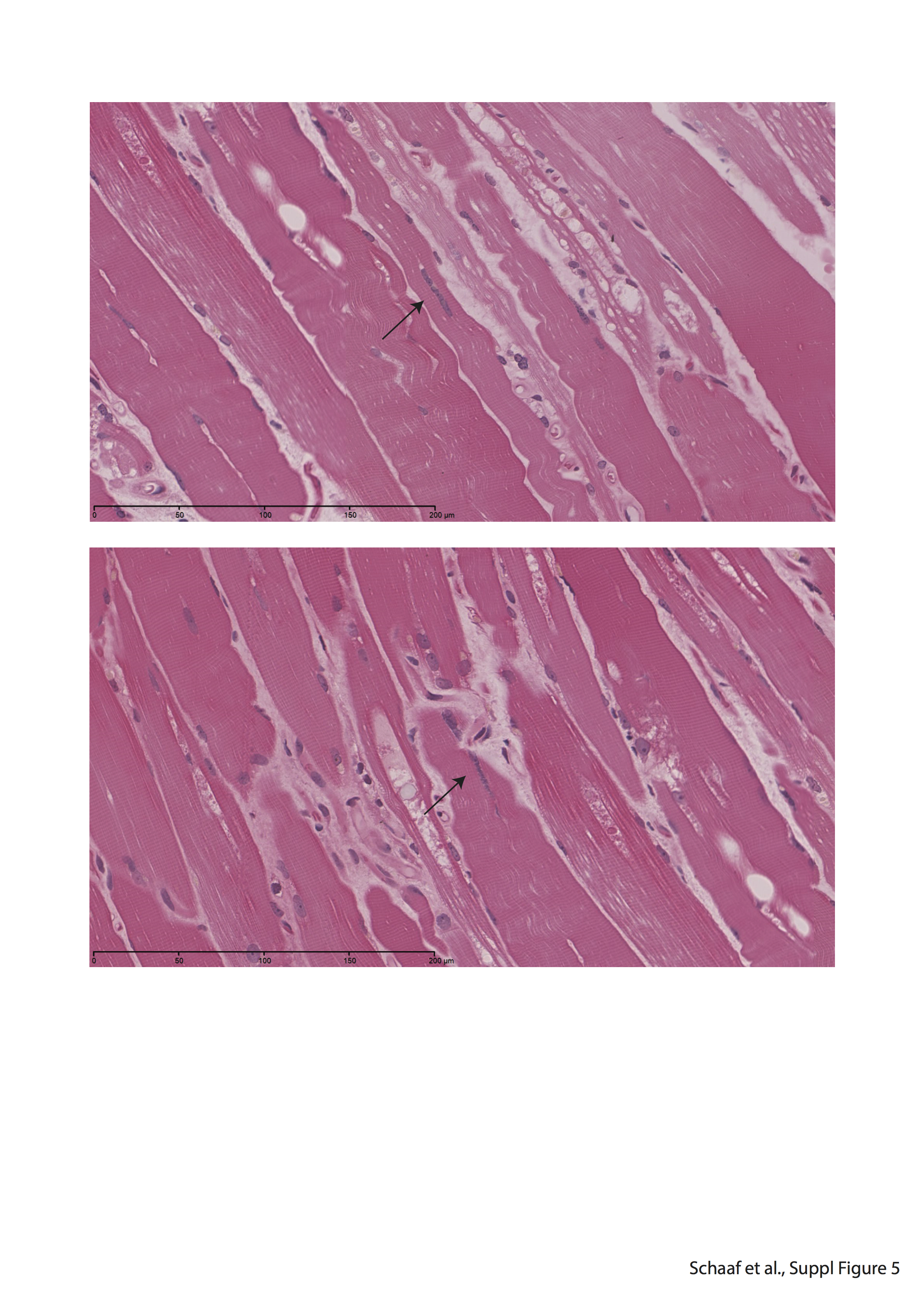

Supplement: Additional file 6: Figure S5. — Examples of muscle fibers that show evidence of recent muscle regeneration. The arrows point to centrally located arrays of nuclei. The two photographs were derived from a muscle biopsy of a severely affected Pompe patient. (JPEG 4375 kb) [file 40478_2015_243_MOESM6_ESM.jpg]
